# Chitosan elicitation enhances biomass and secondary metabolite production in *Carlina acaulis* L.

**DOI:** 10.1038/s41598-025-07085-4

**Published:** 2025-07-02

**Authors:** Maciej Strzemski, Sławomir Dresler, Barbara Hawrylak-Nowak, Przemysław Tkaczyk, Magdalena Kulinowska, Marcin Feldo, Filippo Maggi, Agnieszka Hanaka

**Affiliations:** 1 Department of Analytical Chemistry, Medical University of Lublin, Chodźki 4a Street, 20-093 Lublin, Poland; 2 Department of Plant Physiology and Biophysics, Institute of Biological Sciences, Faculty of Biology and Biotechnology, Maria Curie-Sklodowska University, Akademicka 19 Street, 20-033 Lublin, Poland; 3 Department of Botany and Plant Physiology, Faculty of Environmental Biology, University of Life Sciences in Lublin, Akademicka 15 Street, 20-950 Lublin, Poland; 4 Department of Agricultural and Environmental Chemistry, University of Life Sciences in Lublin, Akademicka 15 Street, 20-950 Lublin, Poland; 5 Department of Vascular Surgery, Medical University of Lublin, Staszica 11 St, 20-081 Lublin, Poland; 6 Chemistry Interdisciplinary Project (ChIP) Research Center, School of Pharmacy, University of Camerino, via Madonna delle Carceri, 62032 Camerino, Italy

**Keywords:** Signaling molecule, Elicitors, Carlina oxide, Chlorogenic acids, Schaftosides, Triterpenic acids, Biotechnology, Plant sciences

## Abstract

*Carlina acaulis* L. (Asteraceae) is a medicinal plant with a long history of use as an herbal remedy. Recently, this species attracted the interest of agrochemical companies due to the presence of valuable biopesticides among which the most promising is carlina oxide (COx). To stimulate the biosynthesis of its specialised metabolites by elicitation, a polysaccharide – chitosan was supplemented as chitosan lactate (ChL). The aim was to evaluate the effects of ChL soil and foliar application on the *C. acaulis* biomass, photosynthetic efficiency, and levels of the COx, chlorogenic acids, protocatechuic acid, schaftosides, and triterpenic acids. ChL has been shown to have no effect on shoot growth while considerably increasing root biomass. It improved photosynthetic efficiency and enhanced levels of specialised metabolites. Soil application of ChL significantly increased chlorogenic and neochlorogenic acid content in leaves, by approximately 330% and 70%, respectively compared to controls. In contrast, foliar application more strongly increased COx levels in roots (55% for foliar vs. 16% for soil treatment). This study has demonstrated that the application of ChL can be used as an inexpensive and accessible way to boost the production of valuable compounds in *C. acaulis*.

**Supplementary Information:**

The online version contains supplementary material available at 10.1038/s41598-025-07085-4.

## Introduction

*Carlina acaulis* L. is a monocarpic perennial species of the Asteraceae family, which is distributed in Central and South Europe, mainly in mountainous areas^1^. The occurrence of this species in the lowlands is rare, but plant material can be obtain in agronomic cultures, either in the field cultivation^2^hydroponically^3–5^ or in vitro^5–7^. This is particularly important because *C. acaulis* plants are a rich source of many valuable chemical compounds, including triterpenes, mainly oleanolic (OA) and ursolic (UA) acids^2,8,9^flavonoids, mainly schaftosides and orientins^10–12^chlorogenic acids (ChAs)^5,12–14^inulin^15,16^ and the polyacetylene carlina oxide (COx)^16–18^. The biological activity of COx is currently the subject of very intensive study. This compound has demonstrated strong antibacterial^18–20^antifungal^18,19^ and antiviral (against SARS-CoV-2)^21^ properties. In addition, COx has been shown to exhibit cytotoxic activity and induce apoptosis in cancer cells^22,23^. Equally interesting is the high activity of COx as a natural insecticide^24^. Indeed, it has been shown to be an effective agent for controlling many pests that affect agricultural production and food stored products, e.g. *Lobesia botrana* Denis & Schiffermüller^25^*Prostephanus truncatus* Horn^26^*Tribolium castaneum* Herbst, *Tribolium confusum* Jacquelin du Val and *Tenebrio molitor* L.^27^. *Acarus siro* L., *Alphitobius diaperinus* Panzer, *Oryzaephilus surinamensis* L., *Rhyzopertha dominica* F., *Sitophilus oryzae* L^28^. as well as insect vectors of medical importance^29,30^ such as *Musca domestica* L. and *Culex quinquefasciatus* Say. Therefore, it is evident that understanding the influence of environmetal factors on plant growth and phytochemistry is valuable for optimising cultivation practices and obtaining the highest-quality yield, especially for rarely cultivated species.

The properties of COx support the hypothesis that it acts as an allelochemical involved in plant-insect interactions. Confirmation of this hypothesis would not only clarify the physiological role of COx in the functioning of *Carlina* plants in the environment, but also enable the effective induction of its biosynthesis and accumulation in plant material, thereby enhancing the quality of raw materials. Verification of the aforementioned theory appears possible through the use of chitosan. Chitosan is a polysaccharide derived from the partial deacylation of chitin, which is a component of insect exoskeletons and fungal cell walls. It can be regarded as an effective elicitor of the plant defence response through the controlled induction of eustress^31–33^. The positive effect of chitosan on the biosynthesis and accumulation of products of specialized plant metabolism, including polyphenols and terpenes, has been demonstrated repeatedly^32,34–36^. This can have a beneficial effect on plant resistance to pathogens and pests. Therefore, the use of chitosan can not only have a positive impact on the quality of the raw material in terms of the source of phytochemicals, but it can also contribute to sustainable crop production^37^. In this study, we used chitosan lactate (ChL), a water-soluble derivative of chitosan that has been shown to be more easily absorbed and to elicit a stronger response than conventional chitosan^36^. Unlike conventional chitosan, which requires acidification to dissolve and may exhibit limited bioactivity in certain systems, ChL dissolves readily in water and can be applied directly via foliar spraying or to the soil.

In this study, we tested the hypothesis for the first time that soil and foliar applications of ChL have beneficial effects on the *C. acaulis* biomass, photosynthetic efficiency, and the content of COx, ChAs, protocatechuic acid, schaftosides, and triterpenic acids.

## Materials and methods

### Plant material, growth conditions, and experimental design

**Fig. 1:**
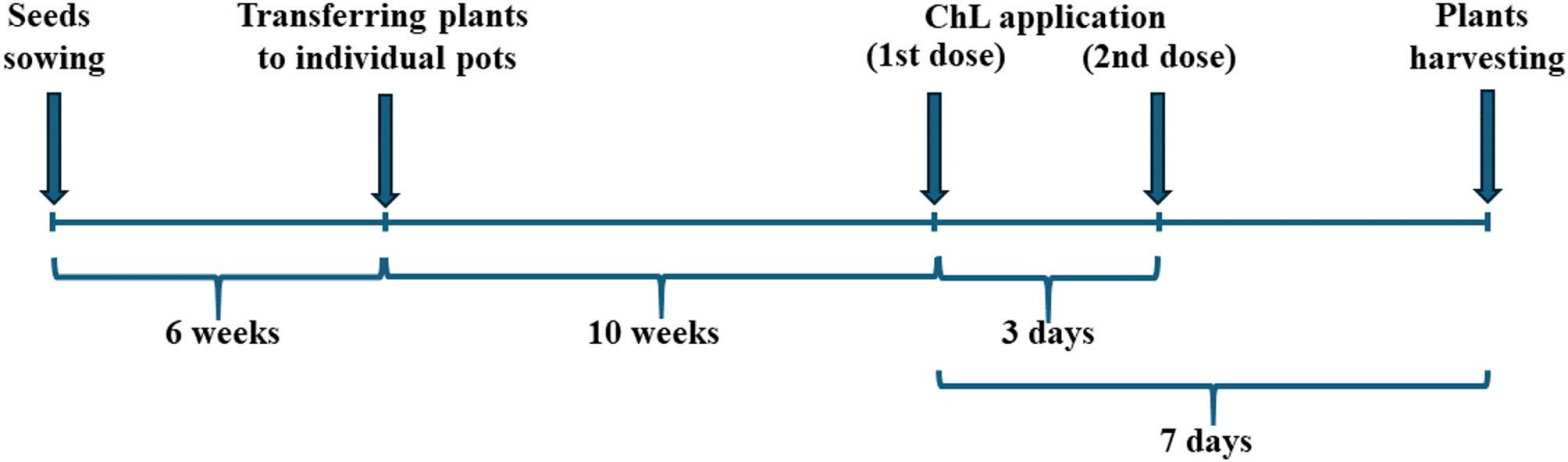
The experimental design scheme.

*Carlina acaulis* L. seeds, obtained from the Botanical Garden of Maria Curie-Skłodowska University (UMCS) in Lublin, Poland (voucher specimen no. 2005 A) were germinated on the standard garden soil. The 6-week-old plants were transferred to pots (one per pot) filled with a mixture of garden soil and vermiculite (1:1, v/v) and cultivated for 10 weeks. Then, the plants were divided into three groups (10 plants per treatment): (I) control, (II) cultivated with ChL applied to the soil and vermiculite mixture (soil ChL); (III) cultivated with ChL applied as foliar spray (foliar ChL). Chitosan lactate was administered twice at a 3-day interval (the first dose immediately after 10 weeks and the second dose after a further 3 days). This form of chitosan was chosen for the study because it is well soluble in water and effectively induces the metabolic response of plants^35^. Chitosan lactate was applied twice at an interval of three days to enhance elicitation efficiency and ensure a sustained metabolic response. This approach is based on the concept of priming, whereby an initial stimulus prepares the plant for a more robust reaction to subsequent exposure^38,39^. The second application was performed at a time when the initial response was expected to decline, thereby reinforcing the signalling cascade and promoting the prolonged accumulation of secondary metabolites. The plants were harvested 7 days after the first dose of ChL was applied (Fig. 1).

Chitosan lactate was applied as a water solution (200 mg in 1 L of H_2_O and 10 mL per 1 plant). A concentration of 200 mg/L of ChL was selected based on previous studies which demonstrated its efficacy in eliciting metabolic responses in medicinal plants^35^. This concentration was slightly higher than that used earlier^35^ due to the xeromorphic features of *C*. *acaulis* leaves, which may hinder ChL penetration. The volume applied during foliar spraying (10 mL per plant) was optimised to fully saturate the leaf surface without runoff into the soil substrate, ensuring uniform and targeted exposure. For comparative purposes, the same dose was introduced directly into the soil substrate, ensuring equivalent ChL delivery regardless of the application method. The control plants were treated with water at the same frequency and in the same volume, regardless the treatment. The plants were grown in the growth chamber in the stable conditions of photoperiod (16 h day/ 8 h night), temperature (25 °C day/18°C night), photosynthetic photon flux density (150 µmol m^− 2^ s^− 1^), and relative humidity (60–65%). After being harvested, the plants were separated into leaves and roots. The roots were washed with deionized water. The fresh biomass of leaves and roots was determined by weighing. The fresh rosettes were used for measurements of chlorophyll fluorescence. One part of the plant material was frozen at − 80 °C immediately after collection for the proline assay, while the other part was air-dried for metabolite determination.

### Reference standards and chemicals

Chitosan lactate (deacetylation degree of 80–95%) was purchased from Heppe Medical Chitosan (Halle, Germany). Oleanolic (OA ≥ 97.0%), ursolic (UA ≥ 90.0%), chlorogenic (≥ 95.0%), neochlorogenic (≥ 98.0%), 3,5-di-caffeoylquinic (3,5CQA) (≥ 95.0%), protocatechuic (≥ 98.0%) acids; schaftoside (≥ 95.0%), and isoschaftoside (≥ 90.0%) were purchased from Sigma-Aldrich (St. Louis, MO, USA). The COx (96.2% purity) was obtained by distillation of *C. acaulis* roots in the Deryng apparatus. The identity and purity of the compound were confirmed in accordance with a previously established methodology^40^. HPLC-grade acetonitrile (ACN), HPLC-grade methanol, trifluoroacetic acid (TFA ≥ 99.0%) and phosphoric acid (H_3_PO_4_ ≥ 85% in H_2_O) were from Merck (Darmstadt, Germany). Water was purified by ULTRAPURE Milli-pore Direct-Q^®^3UV–R system (Merck Millipore, Billerica, Massachusetts, USA).

### Measurement of chlorophyll fluorescence

Chlorophyll fluorescence was performed with a FluorCam 800-MF fluorimeter (Photon Systems Instruments, Czech Republic) in a pulse-amplitude modulated mode (PAM) as was presented in Rysiak et al.^41^. The fluorescence signal emitted by chlorophyll was captured by a CCD camera with an F1.6 (4–10 mm) objective. To relax the reaction centers, the leaves were darkened for 30 min before measurement. The following parameters of chlorophyll fluorescence were obtained: minimal fluorescence (F0), maximal fluorescence (Fm), the maximum quantum efficiency of photosystem II (PSII) photochemistry in the dark-adapted state (Fv/Fm), non-photochemical quenching (qN), photochemical quenching of fluorescence (qP), and vitality index (Rfd). Images were processed using the FluorCam7 software (Photon Systems Instruments, Czech Republic).

### Sample Preparation for determination of specialized metabolite

The plant material was divided into roots and leaves. For chemical analyses the samples were air-dried, pulverized and accurately weighted (0.5000 g). Each sample was extracted four times with methanol (4 × 5 mL) using an ultrasonic bath (4 × 15 min). The obtained extracts were combined to 25 mL in volumetric flasks. Prior to chromatographic analysis, the extracts were centrifuged and filtered through a 0.25 μm nylon membrane filter Millipore (Merck Millipore, Billerica, Massachusetts, USA).

### Determination of secondary metabolites and proline assay

The products of plant specialized metabolism were analyzed using EliteLaChrom chromatograph with UV-VIS PDA detector and EZChrom Elite software (Merck, Darmstadt, Germany). The identity of the analytes was confirmed by comparing their retention times and UV-VIS absorption spectra with those of the corresponding standards. The metabolite content was determined using previously published methodologies (COx^16^; ChAs, protocatechuic acid, and schaftosides^42^; OA and UA^8^) and expressed in mg/g dry weight (DW).

The concentration of free proline, as a reliable indicator of environmental stress, was determined spectrophotometrically (Cecil CE 9500, Cecil Instruments, Cambridge, UK) according to the standard method of Bates et al.^43^ and expressed in µg/g fresh weight (FW).

### Statistical analysis

The experiment was conducted in a completely randomized system with one factor at three levels (control, soil ChL, leaves ChL). A minimum of 4 individual pots, with 1 plant in each pot, were grown for each treatment. The entire experiment was repeated 2 times under the same plant growth conditions. The assumptions of normality of distribution and homoscedasticity of variance were tested using the Shapiro-Wilk and Levene tests, respectively. One-way ANOVA followed by Tukey’s post-hoc test was used to evaluate the significance of differences (*p* < 0.05) between treatments. Statistical analyses were performed using Statistica ver. 13.3 (TIBCO Software Inc. 2017, Palo Alto, CA, USA).

## Results and discussion

### Impact of chitosan on the growth, free proline accumulation, and parameters of chlorophyll fluorescence

**Fig. 2:**
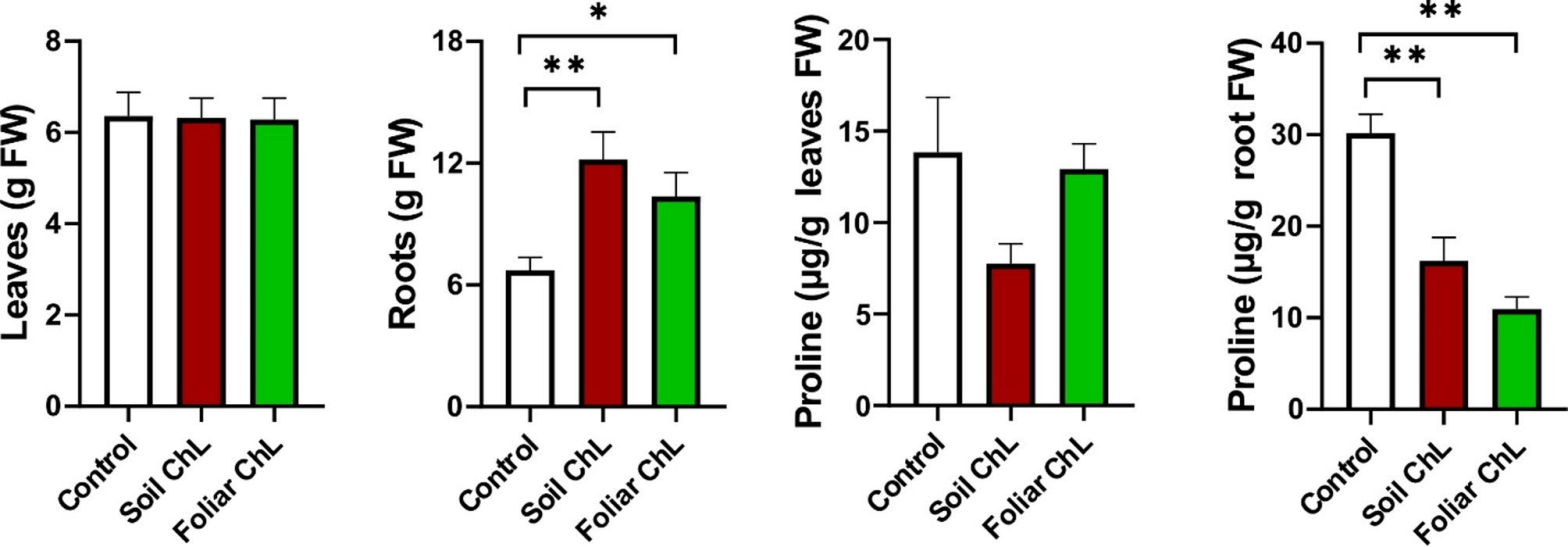
Changes in the fresh weight (FW) of leaves (**a**) and roots (**b**) of *Carlina acaulis* and the content of free proline (**c**,**d**) after the application of chitosan lactate (ChL). Data are means + SE (*n* = 8 for biomass data, *n* = 3 for proline data). *p* < 0.05 (*), *p* < 0.01 (**).

The FW of *C. acaulis* leaves did not change after ChL treatment, regardless of the application method (Fig. 2, Fig. S1). On the other hand, ChL application had a very strong stimulatory effect on root yield, particularly when the plants were irrigated with ChL. In this case, the root weight of an individual plant increased approximately twofold, compared to the control plants. These findings are consistent with previous studies, which indicate that foliar ChL application typically does not affect the shoot FW of certain herbs, i.e. *Ocimum basilicum* L. and *Melissa officinalis* L^34^. Conversly, ChL has also been shown to increase plant growth in some studies, particularly with regard to roots^44^. As multi-directional compounds, chitosan and its derivatives, such as ChL, can modify plant growth parameters and support yield by influencing cell elongation and division, enhancing the uptake of essential nutrients and/or stimulating protein synthesis^45^.

In addition, chitosan provides a signal for the induction of plant immunity during biotic stress by activating secondary signalling pathways in plant cells through binding to specific receptors and activating secondary messengers (Ca^2+^, NO, reactive oxygen species and transcription factors), which are critical for the modification of metabolism and the induction of biochemical defence responses^33^. Given the signalling role of chitosan in stress responses, we interested how it could modify the level of free proline. This proteinogenic amino acid has osmoprotective and antioxidant properties and its accumulation is not limited to alleviating stress, but also modifies plant cell growth and differentiation under non-stress conditions^46^. We found that there were no changes in leaf free proline content, whereas root proline accumulation decreased about twofold after ChL irrigation and by around threefold after foliar application of ChL (Fig. 2). This result was unexpected given that earlier studies had revealed increased proline accumulation in leaves following ChL treatment. However, those experiments focused exclusively on leaf tissues and did not examine root proline content^47^. Some other studies also indicate that foliar applied chitosan may enhance proline accumulation in leaves^48^. There is limited direct evidence regarding the effect of chitosan on the accumulation of free proline under non-stress conditions. Most of the available studies have examined proline levels in the context of chitosan-induced stress resistance, in which increased proline content is considered to be part of the protective biochemical response. However, Zhang et al.^49^ found that enhanced leaf biomass production under non-stress conditions was accompanied by a reduction in proline content in a study investigating the effects of chitooligosaccharides (water-soluble degradation products of chitosan).

It has been suggested that in normal osmotic conditions, free proline accumulation and cell growth are inversely correlated, but these studies were carried out in yeast^50^. Therefore, it is reasonable to assume that the increased root biomass under ChL treatment may be related, at least in part, to a lower concentration of free proline in root cells. However, the reasons why and how the presence of ChL reduces the level of this amino acid in the roots remain unclear and require further insight into biochemical pathways. Some scientists suggest that free proline is incorporated into structural cell wall proteins, such as proline-rich proteins (PRPs), which are known to play a role in cell wall reinforcement and remodelling during growth or in response to signalling molecules, such as elicitors^51^. In addition, the proline cycle is coupled to the shikimic acid pathway, which may alter the biosynthesis of secondary metabolites, particularly aromatic compounds^52^. This issue is relevant in the context of the metabolites we analysed in *C*. *acaulis* leaves and roots. Furthermore, we observed no visible symptoms of stress (e.g. wilting or chlorosis), and the stable or reduced levels of proline in ChL-treated plants suggest that no water stress occurred.

**Fig. 3:**
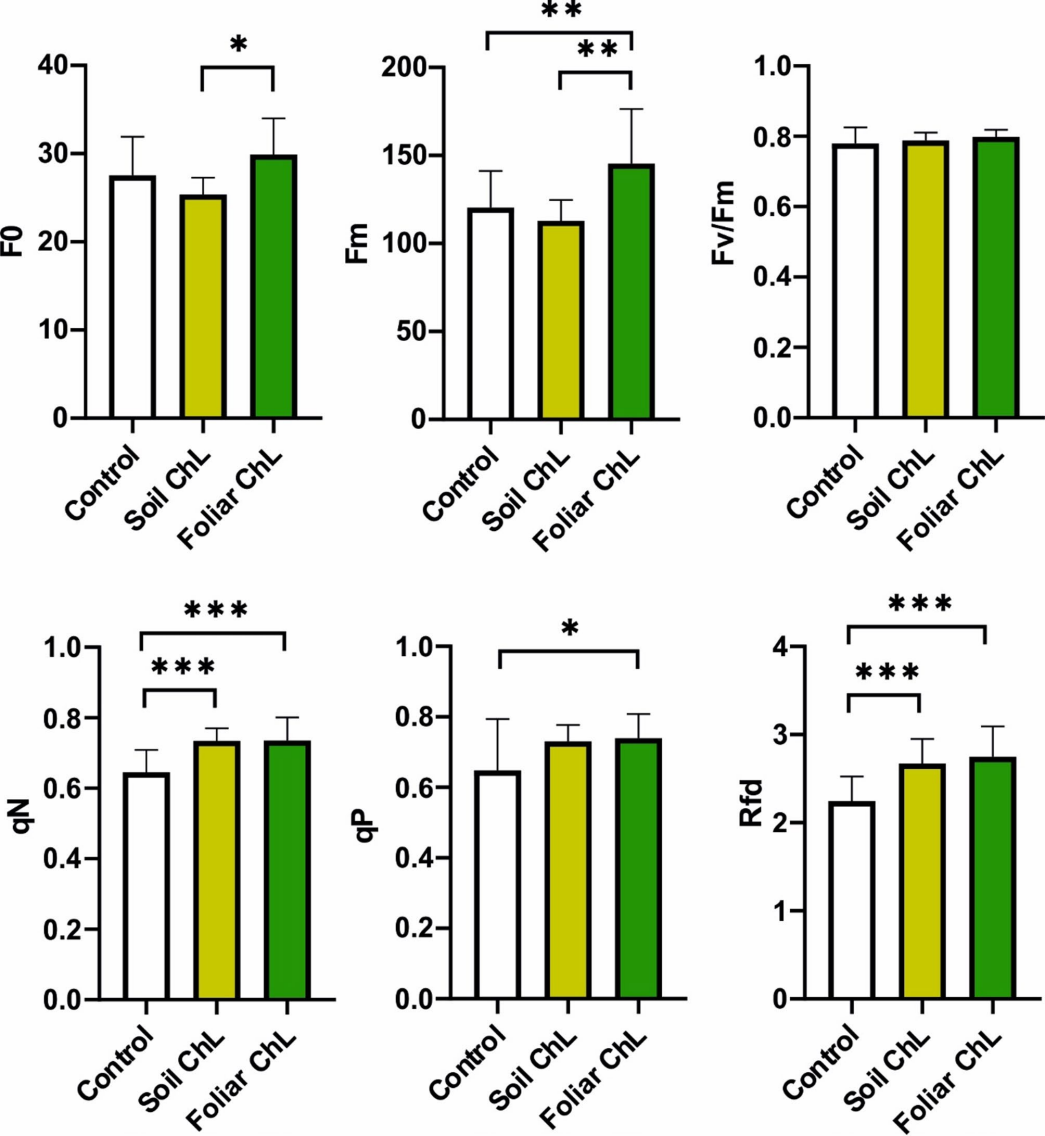
Parameters of chlorophyll fluorescence: (**a**) F0—minimal fluorescence; (**b**) Fm—maximal fluorescence; (**c**) Fv/Fm—the maximum quantum efficiency of PSII photochemistry in the dark-adapted state; (**d**) qN—non-photochemical quenching; (**e**) qP—photochemical quenching of fluorescence; (**f**) Rfd—vitality index (Rfd) in *Carlina acaulis* after the application of chitosan lactate (ChL). Data are means + SE (*n* = 8). *p* < 0.05 (*), *p* < 0.01 (**), *p* < 0.001 (***).

The parameters of chlorophyll fluorescence exhibited considerable sensitivity to the treatments applied (Fig. 3, Fig. S2). The F0 values changed significantly between the type of ChL application (soil vs. foliar), but not in comparison to the control plants (Fig. 3a). A similar effect was determined in *Lallemantia iberica* after foliar treatement with chitosan^53^. The lack of increase in F0 indicates the absence of photoinhibitory damage^54^ upon ChL treatments. Our results indicated that foliar ChL increased the Fm value in the *C. acaulis* leaves, compared to both the soil ChL-treated plants and control plants (Fig. 3b). This phenomenon is in contrast to the findings of Hawrylak-Nowak et al.^34^who demonstrated that a foliar spray with ChL had no effect on Fm in *O. basilicum* and *M. officinalis.* The increase in the Fm parameter can be explained by the dissipation of the captured energy in the form of fluorescence^55^. The qP values achieved in our research differed between the control and the foliar ChL application variants (Fig. 3e).

The qP parameter informs us about the involvement of open PSII reaction centres^54^. The increase in qN following ChL application (both soil and foliar) (Fig. 3d) may suggest changes in heat dissipation^54^ and carotenoid conversion in the xanthophyll cycle^56^.

The Rfd parameter value (Fig. 3f), which represents a trend similar to that of qN (Fig. 3d), increased between the control and both types of ChL application. In plants subjected to stress conditions, Rfd values are generally reduced^41^. Consequently, the significantly higher values of Rfd observed in our experiment, following the application of both types of ChL treatment indicate that the photosynthetic apparatus is functioning more effectively than that of the control group.

The only parameter that did not show a difference among treatments was Fv/Fm (maximum quantum efficiency of PSII) (Fig. 3c), which is in accordance with the results of Paschalidis et al.^57^ on *Origanum dictamnus* L. subjected to various methods of fertilizers application (foliar and soil). Furthermore, similar effects on this parameter were observed in *Phaseolus coccineus* L. under Cu stress^56^and on *O. basilicum* and *M. officinalis* following foliar ChL application^34^. The unaltered levels of Fv/Fm after ChL application suggest the absence of oxidative or photoinhibitory effects on PSII^58^. Other experiments demonstrated that foliar (compared with root) application of chitosan (with deacetylation degree of 63.5%) to *Talinum patens* resulted in reduction in the Fv/Fm value, with no change observed in the qP and qN values^59^.

The chlorophyll fluorescence parameters measured in our experiment provide a useful approach for assessing the photosynthetic functionality of *C. acaulis*. However, it is difficult to differentiate between the soil and foliar application of ChL on the basis of these parameters. In general, ChL stimulated photosynthesis, which was positively associated with leaf gas exchange and hydration status in *Silybum marianum* (L.) Gaertn^60^. Hovewer, it is worth noting that the effect of foliar-applied ChL is both time- and dose-dependent, as presented by El Amerany et al.^61^ in relation to the Fv/Fm values in tomato. Moreover, the outcome may also depend on the plant ecotype^60^.

### Impact of chitosan on the secondary metabolites content in Carlina leaves

**Fig. 4:**
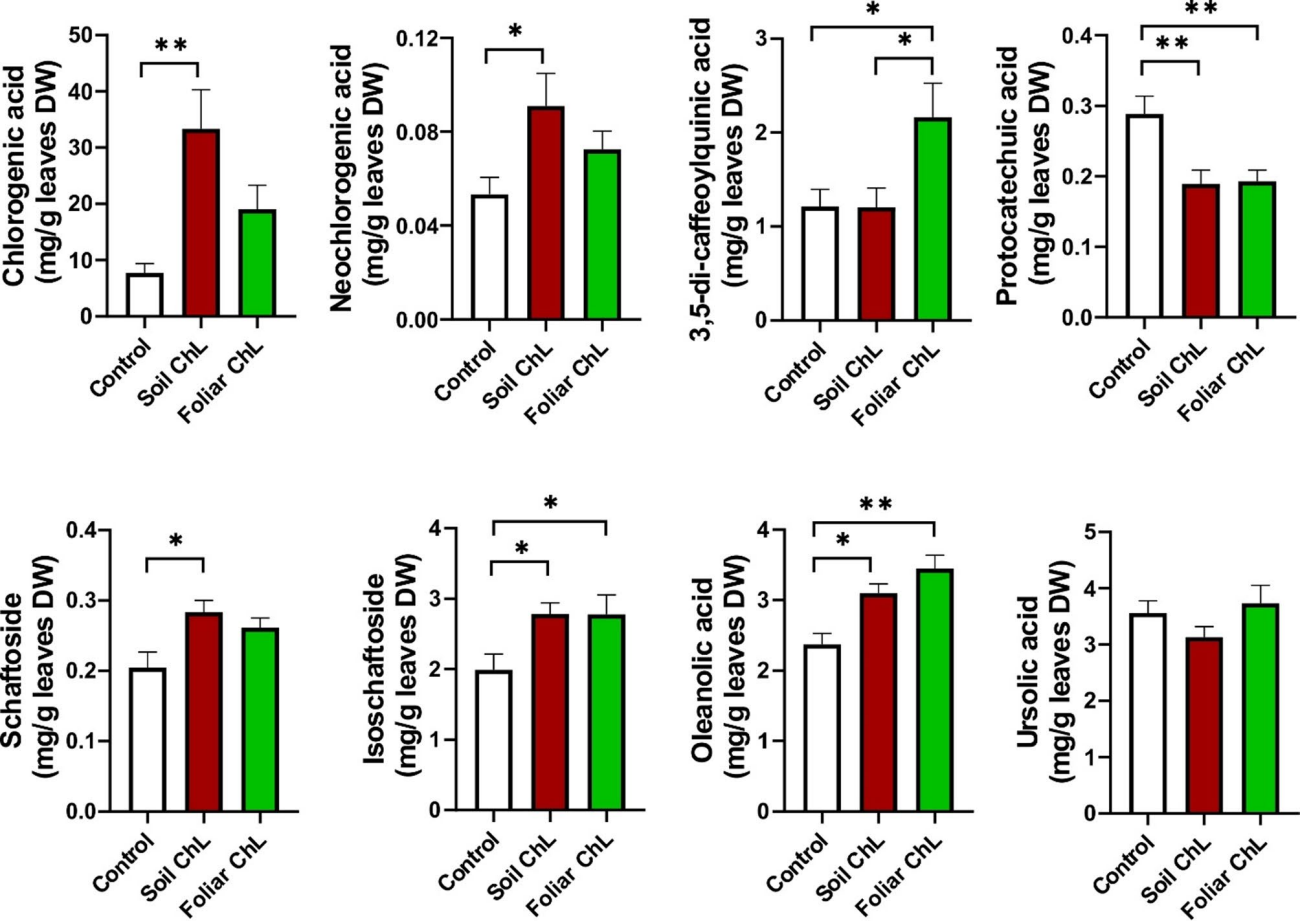
Effects of chitiosan lactate (ChL) on phenolic acids (**a**–**d**), schaftosides (**e**,**f**) and triterpenic acids (**g**,**h**) content in the dry weight (DW) leaves of *Carlina acaulis*. Data are means + SE (*n* = 8). *p* < 0.05 (*), *p* < 0.01 (**).

Chromatographic analysis of extracts obtained from *C. acaulis* leaves revealed the presence of chlorogenic acids, protocatechuic acid, schaftosides, and two triterpenes: OA and UA. The content of each analyte is shown in Fig. 4.

The dominant compound was ChA, with contents of approximately 8, 19, and 33 mg/g DW in control leaves, ChL-sprayed, and ChL-irrigated plants, respectively (Fig. 4a). Thus, foliar application of ChL resulted in a more than twofold increase in ChA content, while soil application resulted in a more than threefold increase. A similar trend was observed for neochlorogenic acid, with a significant increase in its level after soil application of ChL (Fig. 4b). However, the low content of this acid (ca. 0.05–0.1 mg/g DW) suggests that *C. acaulis* is not a suitable raw material for its isolation. Interestingly, soil application of ChL did not increase the level of 3,5-CQA, while ChL foliar application doubled the content of this depside compared with control and soil-treated plants (Fig. 4c). A positive effect of ChL on the accumulation of depsides (including chlorogenic acid and neochlorogenic acid by 42 and 37%, respectively) in the leaves of *Plectranthus amboinicus* (Lour.) Spreng. was demonstrated by Stasińska-Jakubas et al.^36^. ChL also increased depsides accumulation in the leaves of *M. officinalis* and *O. basilicum*^34^. These data are consistent with our observations in *C. acaulis* and confirm the important role of chlorogenic acids in plant responses to stress induced by insect feeding or fungal infection.

Both soil and foliar application of ChL significantly reduced protocatechuic acid level, regardless of the method of ChL application. ChL-treated plants contained approximately 30% less protocatechuic acid than control plants (Fig. 4d). To the best of our knowledge, the effect of ChL on protocatechuic acid content in plants has not yet been studied. However, there is data indicating the role of this compound in insect sclerotization and suggesting that it may be the source of insect diet^62^. Limiting the accumulation of protocatechuic acid in plant tissues would suggest that plants have a highly advanced defence mechanism against insects. However, this reaction could also explain the reduction in the content of this compound in *C. acaulis* leaves that we observed.

ChL (supplied *via* the soil or foliage) induced a significant increase in flavonoid di-C-glycosides content (schaftoside and isoschaftoside), although the method of application was generally not significant (Fig. 4e-f). An increase in flavonoid levels under the influence of chitosan was also observed by Jiao et al.^63^ in *Isatis tinctoria* L. hairy root cultures. Interestingly, no clear trend was observed in the level of pentacyclic triterpenes. The content of OA was significantly higher in ChL-treated plants, with the highest content found in the foliar application variant (Fig. 4g). In contrast to the changes in OA content, no significant differences in UA content were detected (Fig. 4h). The increase in OA content observed in our study appears to follow the trend identified by Lucini et al.^37^. These authors demonstrated that chitosan induces the biosynthesis of pentacyclic triterpenes in grapevine fruits. They found that ursolate and oleanolate contents increased by 1.25 and 1.47 times, respectively^37^.

The metabolite contents determined in the collected material allow us to conclude that the cultivation of *C. acaulis* in a soil/vermiculite substrate provides plant material with a composition comparable to that obtained from field cultivation and much richer compared with hydroponic cultivation, in which the roots were placed directly in the liquid medium. In addition, the use of ChL enriches the material in metabolites compared with material obtained from field-grown plants. For example, Strzemski et al.^5^ found that *C. acaulis* leaves from field and hydroponic cultivation, contained 10.21 and 2.82 mg/g of chlorogenic acid, 0.91 and 0.77 mg/g of 3,5-QCA, 2.05 and 1.20 mg/g of OA, 5.57 and 3.64 mg/g of UA, respectively. Thus, cultivation in the soil/vermiculite substrate resulted in plants with a similar chlorogenic acid content to that of field-grown plants, whereas substrate treatment with ChL yielded material that was three times richer in this compound. In turn, foliar application of ChL resulted in leaves containing twice the amount of 3,5-QCA compared with field cultivation. The level of OA in the leaves of plants treated with foliar ChL was also higher than in plants grown in the field. Only in the case of UA, cultivation in the soil/vermiculite substrate and application of ChL did not produce the desired results. The leaves of the plants obtained in this experiment contained similar amounts of UA to the leaves of plants grown in hydroponics with the fully submerged roots in the nutrient solution^5^. However, Dresler et al.^3^ showed that the UA content of *C. acaulis* shoots grown in hydroponics for 90 days was approximately 1 mg/g DW with the OA content of less than 0.5 mg/g DW. From this perspective, the results obtained with soil/vermiculite cultivation are promising in terms of developing efficient agronomic practices for *C. acaulis*.

### Impact of chitosan on the secondary metobolites content in Carlina roots

**Fig. 5:**
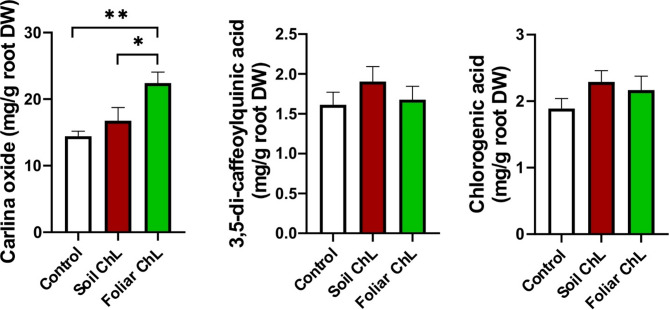
Effect of chitiosan lactate (ChL) on carlina oxide (**a**) and chlorogenic acids (**b**,**c**) contents in the dry weight (DW) roots of *Carlina acaulis*. Data are means + SE (*n* = 3). *p* < 0.05 (*), *p* < 0.01 (**).

Two depsides (chlorogenic acid and 3,5-CQA) and COx were determined in the root extracts obtained in this study (Fig. 5).

In contrast to the results obtained for leaf extracts, no significant differences were found in the depsides content in root extracts from control and ChL-treated plants (Fig. 5b-c). These results were consistent with those previously reported by Strzemski et al.^5^ for plants grown in both field and hydroponic conditions. Nevertheless, the most interesting compound present in *C. acaulis* roots is COx. This compound has been extensively studied for its antimicrobial^20^ and insecticidal activities^24^. Plants sprayed with ChL exhibited a 36% increase in COx content compared with control plants, while those irrigated with ChL exhibited a 26% increase (Fig. 5a). Although the expected effect of watering the plants with ChL was only partially achieved, foliar application of ChL appears to be an effective method of obtaining COx-rich raw material. The use of ChL to stimulate COx biosynthesis is beneficial since it considerably increases root biomass, in which this compound accumulates, allowing a greater amount of COx to be obtained per plant. This result is particularly important from the industrial perspective, given the use of *C. acaulis* raw material in the production of botanical insecticides. Indeed, any sustainable strategy to increase the content of valuable compounds in industrial crops should be carefully considered.

The observed increase in the accumulation of secondary metabolites following the application of ChL may be due to its function as a potent elicitor of plant defence pathways. Due to its water solubility and bioavailability, ChL is easily recognized by plant pattern recognition receptors, triggering early signalling events such as Ca^2+^ influx and the generation of reactive oxygen and nitrogen species, which are considered hallmarks of early defence signalling in plants^31,33^. These signals converge on transcriptional networks that control phenylpropanoid and isoprenoid metabolism, leading to increased biosynthesis of phenolic acids, flavonoids and triterpenes^32,37^. Furthermore, ChL is likely to interact with phytohormonal signalling, particularly the jasmonic and salicylic acid pathways^64^which are known to regulate specialised metabolism. The observed decline in root proline content, accompanied by enhanced phenolic biosynthesis, suggests a potential shift in metabolic flux from nitrogen-rich osmoprotectants towards carbon-based secondary metabolites. Proline biosynthesis and degradation are metabolically linked to the shikimate and phenylpropanoid pathways via glutamate and pyrroline-5-carboxylate, which could explain these changes biochemically^52^. These findings are consistent with a controlled eustress response that enhances biochemical defences without compromising plant growth or photosynthetic performance.

## Conclusions

*Carlina acaulis* is a highly regarded medicinal plant with a long history of use in several European countries. Recently, it has gained attention as a potential industrial crop for the production of biopesticides for use in agriculture and other fields. This study demonstrates for the first time that applying ChL to C. acaulis is an effective method of obtaining a raw material with a high content of the insecticide carlina oxide, along with phenolic acids (excluding protocatechuic acid) and pentacyclic triterpenes. Furthermore, regardless of the application method, ChL does not appear to have any negative impact on plant growth. In fact, in the case of roots, it causes an about twofold increase in biomass. The enhanced accumulation of carlina oxide in the roots of ChL-treated plants may explain the role of this compound in plant physiology as a natural insecticide.

## Electronic supplementary material

Below is the link to the electronic supplementary material.

Supplementary Material 1

## Data Availability

The data required to reproduce these findings are available from the corresponding author (M. Strzemski) upon reasonable request.
