# Supplementary material for: Chitosan elicitation enhances biomass and secondary metabolite production in Carlina acaulis L
Source: Sci Rep. 2025 Jul 2;15:23411. doi: 10.1038/s41598-025-07085-4 (PMC12222969; doi:10.1038/s41598-025-07085-4)
Supplement: Supplementary file 1 — Supplementary Material 1 [file 41598_2025_7085_MOESM1_ESM.docx]

**SUPPLEMENTARY MATERIALS**

**Chitosan elicitation enhances biomass and secondary metabolite production in *Carlina acaulis* L.**

**Maciej Strzemski ^a,^*, Sławomir Dresler ^a,b^, Barbara Hawrylak-Nowak ^c^, Przemysław Tkaczyk ^d^, Magdalena Kulinowska ^a^, Marcin Feldo ^e^, Filippo Maggi ^f^, Agnieszka Hanaka ^b,^***

^a^ Department of Analytical Chemistry, Medical University of Lublin, Chodźki 4a Street, 20-093, Lublin, Poland

^b^ Department of Plant Physiology and Biophysics, Institute of Biological Sciences, Faculty of Biology and Biotechnology, Maria Curie-Sklodowska University, Akademicka 19 Street, 20-033 Lublin, Poland

^c^ Department of Botany and Plant Physiology, Faculty of Environmental Biology, University of Life Sciences in Lublin, Akademicka 15 Street, 20-950 Lublin, Poland

^d^ Department of Agricultural and Environmental Chemistry, University of Life Sciences in Lublin, Akademicka 15 Street, 20-950 Lublin, Poland

^e^ Department of Vascular Surgery, Medical University of Lublin, Staszica 11 St., 20-081 Lublin, Poland

^f^ Chemistry Interdisciplinary Project (ChIP) research center, School of Pharmacy, University of Camerino, via Madonna delle Carceri, 62032 Camerino, Italy

*Corresponding authors

*E-mail addresses:* [maciejstrzemski@umlub.pl](mailto:maciejstrzemski@umlub.pl) (M. Strzemski); [agnieszka.hanaka@mail.umcs.pl](mailto:agnieszka.hanaka@mail.umcs.pl) (A. Hanaka)

Figure S1. Control, soil Chl, and leaves ChL treated *C. acaulis* plants cultivated under controled conditions for 17 weeks.


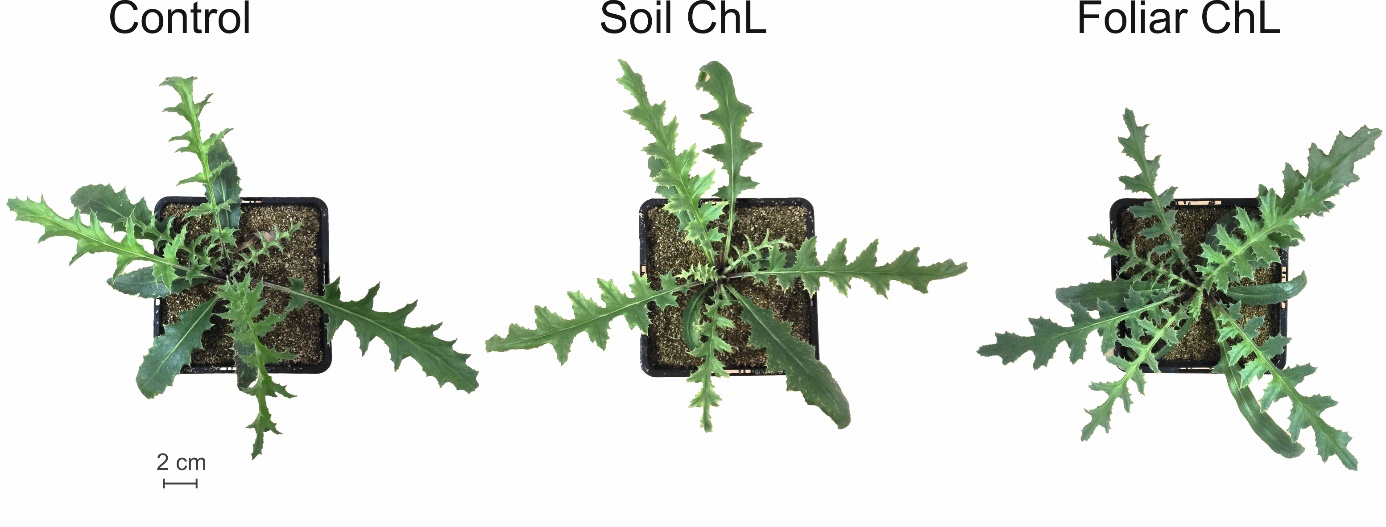


Figure S2. Visualisation of chlorophyll fluorescence parameters in whole *C. acaulis* rosettes.
